# Supplementary material for: Red Blood Cells from Individuals with Lesch–Nyhan Syndrome: Multi-Omics Insights into a Novel S162N Mutation Causing Hypoxanthine-Guanine Phosphoribosyltransferase Deficiency
Source: Antioxidants (Basel). 2023 Aug 31;12(9):1699. doi: 10.3390/antiox12091699 (PMC10525117; doi:10.3390/antiox12091699)
Supplement: Supplementary file 1 [file antioxidants-12-01699-s001.zip › Supplementary Figure S1.pdf]

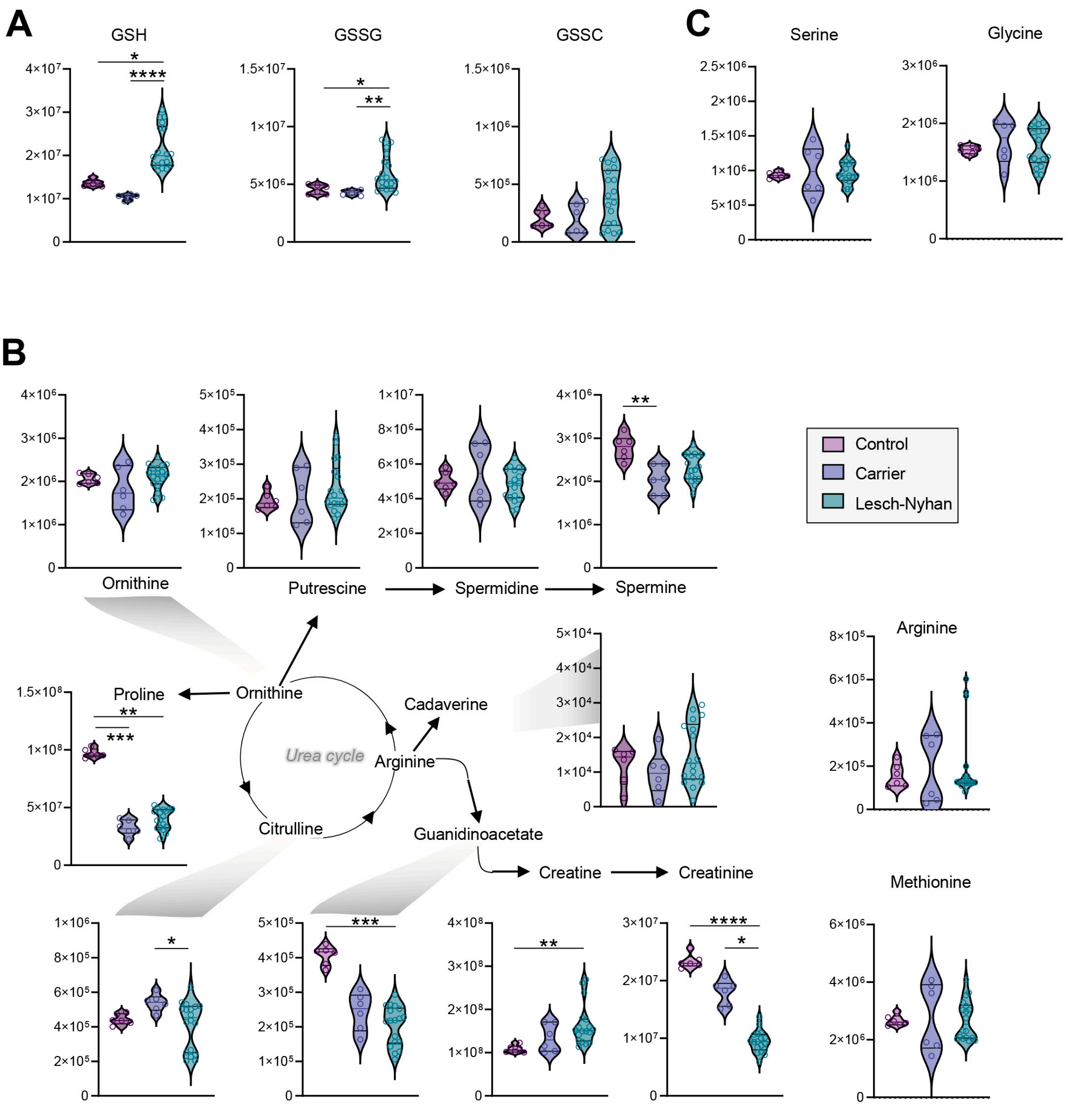

Figure S1. (**A**). Overview of transamination reactions reveals decreased amido-containing amino acids Gln and Asn in LN RBCs and increased carboxylate amino acids Glu and Asp. (**B**). Glutathione pool metabolites. (**C**). Overview of urea cycle/polyamine metabolites. (**A–C**) Data are peak areas (au). Statistics are one-way ANOVA with Kruskal-Wallis multiple comparisons test. * $p < 0.05$, ** $p < 0.01$, *** $p < 0.001$, **** $p < 0.0001$.
